# Sequencing Shorter cfDNA Fragments Decreases the False Negative Rate of Non-invasive Prenatal Testing

**DOI:** 10.3389/fgene.2020.00280

**Published:** 2020-03-26

**Authors:** Ying Xue, Guodong Zhao, Longwei Qiao, Jiafeng Lu, Bin Yu, Ting Wang

**Affiliations:** ^1^The Affiliated Suzhou Hospital of Nanjing Medical University, Suzhou, China; ^2^Suzhou Municipal Hospital, Suzhou, China; ^3^State Key Laboratory of Bioelectronics, School of Biological Science and Medical Engineering, Southeast University, Nanjing, China; ^4^Zhejiang University Kunshan Biotechnology Laboratory, Zhejiang University Kunshan Innovation Institute, Kunshan, China; ^5^Changzhou Maternity and Child Health Care Hospital Affiliated to Nanjing Medical University, Changzhou, China

**Keywords:** NIPT, false negative, shorter cfDNA fragments, size selection, twin pregnancies, confined placental mosaicism

## Abstract

Circulating fetal cell-free DNA (cfDNA) is generally shorter than maternal cfDNA. Size selection of shorter cfDNA in total cfDNA could significantly increase the fetal fraction, but there are few reports of using this method to decrease the false negative rate for NIPT. In this study, nine false negative cases were retrospectively analyzed by NIPT retesting and E-gel based size-selection NIPT and the fetal cfDNA fraction in maternal total cfDNA was evaluated by calculating the proportion of reads from chromosome Y. Fetal placenta karyotypes were confirmed by CNVplex assays to analysis the reasons for false negative cases. Of the 81,601 pregnancies who underwent NIPT, nine false negative cases (0.01%) were found. Of eight retested cases, two (25%) had positive NIPT retest results, and five (62.5%) had positive size-selection NIPT results. For fetal cfDNA fraction, 100% cases had improvement after size-selection NIPT compared with the initial NIPT and retest results, and the fetal cfDNA fraction growth ratio ranged from 99 to 359%. For one twin pregnancy with one T18 fetus, size selection improved the fetal cfDNA fraction to 23.10%, and successfully detected the T18 fetus in NIPT. Placental tissue analysis results for two cases indicated both had confined placental mosaicism (CPM), which was confirmed with size-selection NIPT. In conclusion, size selection can significantly enrich the fetal cfDNA fraction and decrease the false negative rate of NIPT, especially for CPM and twin pregnancies.

## Introduction

Non-invasive prenatal testing (NIPT) is widely used for detecting fetal chromosome trisomies 13, 18, and 21 (T13, T18, and T21) and sex chromosome aneuploidies with high sensitivity and specificity (Porreco et al., 2014; Yu et al., 2017; Zhang et al., 2017). However, this method does have limitations such as maternal malignancies and confined placental mosaicism (CPM) that lead to discordance between the fetal karyotype and NIPT results (Wang et al., 2014; Bianchi et al., 2015), not all possible genetic disorders are detectable, some false positive or negative cases, as well as 3–5% non-reportable results due to low fetal fraction circulating free DNA (cfDNA) (Liehr et al., 2017; Suzumori et al., 2019).

A previous study reported the false negative rate of NIPT for T21 detection was only 0.09%, which is significantly lower than the false positive rate for the condition (Hartwig et al., 2017). However, false negative NIPT results cannot be ignored in clinics and should be given more attention than false positive results because they are associated with more serious consequences and cause more stress to pregnant women and their families.

It is well established that fetal cfDNA is generally shorter than maternal cfDNA in maternal plasma; the main size distribution of maternal DNA is 166 bp, whereas fetal DNA has a smaller 166-bp peak and a relatively prominent 143-bp peak (Lo et al., 2010). Therefore, size selection of shorter cfDNA during DNA extraction or library construction for subsequent cfDNA sequencing analysis could significantly increase the fetal cfDNA fraction (Minarik et al., 2015). Recently, several studies reported using size selection to improve NIPT specificity (Minarik et al., 2015; He et al., 2018), but the application of this method to reanalyze NIPT false negative cases has not been well described.

In this study, we retrospectively analyzed false negative cases in our clinical center using NIPT retesting and E-Gel-based cfDNA size selection to identify factors involved in false negative NIPT results and the feasibility of size selection for decreasing the false negative rate.

## Materials and Methods

### Samples

We assessed the records from 81,601 pregnancies that underwent NIPT from February 2012 to December 2018 in the Center for Reproduction and Genetics at the Affiliated Suzhou Hospital of Nanjing Medical University. The gestational ages for the nine false negative pregnancies at the time of blood collection were 16 to 26 weeks, the maternal ages were 26 to 41 years old, and the paternal ages were 24 to 45. The study was approved by the Institutional Review Board of The Affiliated Suzhou Hospital of Nanjing Medical University. All subjects provided written informed consent prior to participation.

### Sample Processing and DNA Extraction and Sequencing

Ten milliliters of peripheral blood from each pregnant woman was drawn into a K3EDTA Vacuette tube (Becton-Dickinson, Franklin Lakes, NJ, United States), the plasma fractions were then separated in 4 h and cfDNA from maternal plasma was captured on magnetic beads, purified, and concentrated following the manufacturer’s protocol. Purified cfDNA was used for DNA concentration measurement and library construction following the manufacturer’s protocol (Berry Genomics Co., Limited, Beijng, China) (DAAN Gene Co., Ltd, Shenzhen, China). Massively parallel sequencing was performed on the Illumina NextSeq CN500 platform (Illumina, San Diego, CA, United States) and Ion torrent Proton system (Thermo Fisher Scientific, Waltham, MA, United States). All sequencing data were mapped to the hg19 human reference genome, and fetal DNA concentrations were evaluated by calculating the proportion of reads from chromosome Y as previously described (Wang et al., 2016b), and fetal fraction in female pregnancies was not calculated. Male fetal cfDNA fraction was calculated by fo llowing formula.

Fetal⁢cfDNA⁢fraction

 =(%chrY-M⁢F%chrY)F⁢F/(%chrY-A⁢M%chrY)F⁢F

Where %*chrY*_*MF*_ is the Y chromosome percentage of pregnant women carrying a male fetus; % *chrY*_*FF*_ and % *chrY*_*AM*_ are the median of the Y chromosome percentage from pregnant women and three adult males (Wang et al., 2016b).

Fetal aneuploidy status for whole chromosomes was determined by *Z*-scores (normal: −3 < Z < 3).

### Size-Selection NIPT

Size-selection NIPT was processed according to an previously published protocol (He et al., 2018). Briefly, 600 μL plasma from 8 false negative pregnancies was subjected to cfDNA extraction using TIANamp Micro DNA Purification kits (Tiangen Biotech, Beijing, China). The DNA library was constructed with an Ion Plus Fragment Library Kit (Thermo Fisher Scientific). Polymerase chain reaction products were chosen from 197 to 235 bp (insert DNA ranging from 107 to 145 bp) using E-Gel EX 2% Gels (Invitrogen, Carlsbad, CA, United States). The selected fragments were sequenced using the Ion torrent Proton system. The fetal cfDNA fraction after size-selection NIPT in maternal total cfDNA also evaluated by calculating the proportion of reads from chromosome Y as described above (Wang et al., 2016b; Qiao et al., 2019).

### Ultrasound Examinations

Ultrasound screening was performed using the Voluson 730 Expert system (GE Healthcare, Chicago, IL, United States) with a 2–5 MHz transabdominal convex transducer and a three-dimensional (3D) broadband curved array transducer (3D6-2,2–6 MHz) following routine fetal ultrasound scan guidelines [8]. The following sonographic parameters were assessed to estimate fetal biometry and health: biparietal diameter, head circumference, abdominal circumference, femur diaphysis length, and humerus length. Conventional ultrasound scanning was also performed to examine the development of the fetus’ head, face, spine, chest and abdomen, internal organs, and limbs. The placenta, umbilical cord, and amniotic fluid were also examined.

### Chromosome Karyotype Analysis

Pregnant women with positive NIPT results for chromosome aneuploidies or abnormal ultrasound results consented to invasive prenatal diagnosis. Amniocentesis was performed under sterile conditions and ultrasound guidance in our center. The amniocytes and peripheral blood cells were cultured at 37°C. A total of 60 dividing phases were counted using an AI chromosome image analysis system based on the principle of “An International System for Human Cytogenetic Nomenclature, ISCN2013,” and 20 G-banded metaphases from each sample were analyzed in triplicate (Wang et al., 2016a, b).

### Placental Tissue Analysis

Two duplicate biopsy samples from various regions of placental tissue were collected. Karyotype analysis was performed using CNVplex assays following a previously described method (Yang et al., 2016). Placental tissue genomic DNA was extracted with a TIANamp Genomic DNA kit (Qiagen, Hilden, Germany) according to the manufacturer’s instructions. All samples were analyzed by 170 pairs of probes for 24 chromosomes.

### Data Analysis and Statistics

The difference of unique reads, fetal cfDNA fraction, fetal cfDNA fraction growth ration after fetal cfDNA enrichment and the size peak distribution between size-selection NIPT, NIPT retesting, and initial NIPT were used to assess the feasibility of size-selection NIPT decreases the false negative rate.

## Results

### Sample Characteristics

**FIGURE 1:**
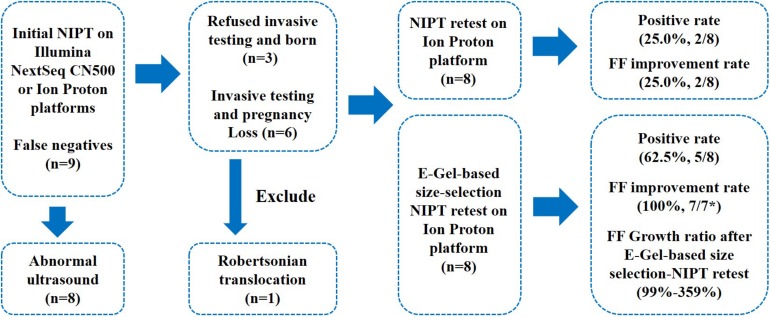
Outcomes of NIPT false negative cases after invasive testing, NIPT retesting, and size-selection NIPT retesting. FF, fetal cfDNA fraction. *FF was evaluated by calculating the proportion of reads from chromosome Y, and case 5 was a female fetus, so the FF could not be calculated.

Of the 81,601 pregnancies who underwent NIPT, only 9 false negative cases (0.01%) were found, including 4 cases of T18 and 5 cases of T21 (1 Robertsonian translocation). The gestational ages at the time of blood collection ranged from 16+ to 26+ weeks. The maternal body mass index (BMI) was ≥25 in three women, and one case was an advanced age twin pregnancy (Figure 1 and Table 1). Abnormal ultrasound results were found in 8 false negative cases during the second trimester. Out of nine false negative cases, 6 pregnancies (66.6%) underwent further invasive testing via amniocentesis after ultrasound examination, and found fetal chromosome aneuploidies. After further careful genetic counseling, these six women selected to terminate pregnancy. The other three cases refused confirmatory diagnosis, even two of them had abnormal ultrasound results and were considered to have fetal chromosome aneuploidies based on neonatal blood karyotyping (Figure 1).

**TABLE 1:** Details of false negative cases and pregnancy results.

**Case**	**MA**	**BMI**	**GW**	**PA**	**Twin pregnancies**	**IVF-ET pregnancies**	**Ultrasound results**
Case 1	26	16.26	18 + 3	28	NO	NO	Nasal insufficiency, left renal sinus splitting
Case 2	33	19.84	16 + 4	35	NO	NO	N/A
Case 3	31	26.57	17 + 3	39	NO	NO	Multiple malformations
Case 4	30	23.15	26 + 4	31	NO	NO	Hypoplastic nasal bone
Case 5	26	17.97	18 + 4	27	NO	NO	Absent nasal bone
Case 6	36	21.29	16 + 1	36	NO	NO	Absent nasal bone
Case 7	28	25.91	18 + 4	29	NO	NO	Multiple malformations: small jaw, cystic dilatation, overlapping fingers on the left hand
Case 8	24	23.05	20 + 4	24	NO	NO	Right ventricular outflow tract and pulmonary stenosis
Case 9	41	26.50	16 + 5	45	YES	YES	Normal left fetus, right fetus with a complex malformation

*BMI, body mass index; GW, gestational age at blood collection; IVF-ET, *in vitro* fertilization-embryo transfer; MA, maternal age; PA, paternal age.*

### Performance of E-Gel-Based cfDNA Size-Selection NIPT

**FIGURE 2:**
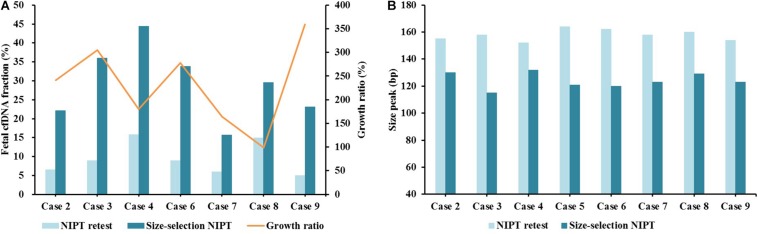
The fetal cfDNA fraction between NIPT retesting and E-Gel-based size-selection NIPT retesting. **(A)** Fetal cfDNA fraction and fraction growth ratio after E-Gel-based size-selection NIPT. **(B)** Size peak distribution.

Of the nine false negative cases, eight were processed with further NIPT retesting and size-selection NIPT retesting. The Robertsonian translocation case was excluded due to without remaining samples. Two (25.0%) cases had positive NIPT retest results (Table 2): one case (case 2) had borderline unique reads (<2 M), while the other one was a pregnancy with CPM (case 7) (Table 3). On size-selection NIPT retesting, five (62.5%) cases were positive, including the two that were positive with NIPT retesting (Table 2). With regard to fetal cfDNA fraction, 100% (7/7) of cases had significant improvement compared with initial NIPT and retesting, and the fetal cfDNA fraction growth ratio ranged from 99 to 359% (Figure 2A). The cfDNA size peaks for NIPT retesting ranged from 152 to 164 bp, and the cfDNA size peak for those cases significantly decreased after size selection (range 115 to 132 bp) (Figure 2B).

**TABLE 2:** The data of initial NIPT, NIPT retesting, and size-selection NIPT retesting.

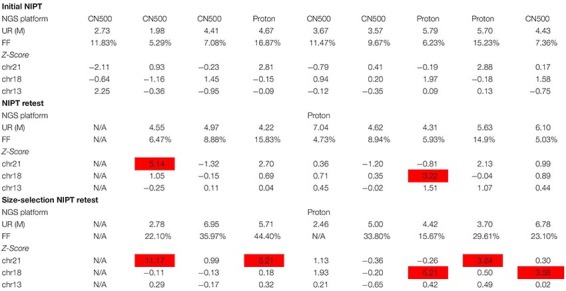

*NGS, next-generation sequencing; NIPT, non-invasive prenatal testing; UR, unique reads; FF, fetal cfDNA fraction. The highlight refer to those values are positive based on the cut-off for *Z-score*.*

**TABLE 3:** The details of fetal karyotypes, placental tissues analysis, pregnancy results, and false negative reasons for false negative cases.

**Case**	**Fetal karyotypes**	**Placental tissue analysis**	**Pregnancy results**	**False negative reasons**
Case 1	46, XY, +21, [rob (21;21) (q10;q10)]	N/A	TOP, male	Uncertain
Case 2	47, XY, +21	N/A	Born, male	Borderline unique reads
Case 3	47, XY, +18	N/A	TOP, male	Uncertain
Case 4	47, XY, +21	N/A	Born, male	Uncertain
Case 5	47, XX, +18	N/A	Born, female	Uncertain
Case 6	47, XY, +21	N/A	TOP, male	Uncertain
Case 7	47, XY, +18	Placental-fetal side: 47, XY, +18 Placental-maternal: 45, XY, −18	TOP, male	CPM
Case 8	47, XY, +21	Placental-fetal side: 46, XY; Placental-maternal side: 46, XY/69, XXY; Umbilical cord: 47, XY, +21	TOP, male	CPM
Case 9	47, XY, +18	N/A	Left: born, male; Right: TOP, male	Twin pregnancy low fetal fraction

*BMI, body mass index; CPM, confined placental mosaicism; N/A, not applicable; TOP, termination of pregnancy.*

### Placental Tissue Analysis of CPM

Placental tissues of two pregnancies were collected and analyzed by CNVplex assays. The results revealed that the placental tissue of case 7 showed discordance between the placental-fetal side (47, XY, +18) and placental-maternal side (45, XY, −18). The placental-maternal side for case 8 had mosaic T21, while the placental-fetal side had a normal karyotype and the umbilical cord had the same karyotype as the fetus (Table 3).

### E-Gel-Based cfDNA Size-Selection NIPT in Twin Pregnancy

Case 9 was conceived with IVF-ET and yielded a twin pregnancy in a patient of advanced age (41 years old) with a BMI of 26.5. The fetal cfDNA fraction on initial NIPT was 7.36% (Table 1 and Figure 2). Ultrasound indicated the left fetus was normal but the right fetus had a complex malformation. Karyotyping validation confirmed the right fetus had chromosomal aneuploidy. After careful genetic counseling, the right male fetus was aborted, and the normal left male fetus was born. On NIPT retesting, the fetal cfDNA fraction of case 9 decreased to 5.03%, and the cfDNA size peak distribution was 154 bp; the case was again missed (Figure 2). After E-Gel-based cfDNA size selection, the fetal cfDNA fraction increased to 23.10% with a cfDNA size peak distribution of 123 bp, which was successfully detected (Figure 2).

## Discussion

In this retrospective study, a total of 81,601 pregnancies were analyzed using next-generation sequencing-based NIPT, and only 0.01% had false negative results, which is in accordance with a previous study (Suzumori et al., 2019). False negative results might lead to the birth of children with trisomies and increase burdens on families. We re-analyzed all nine false negative cases in our center to identify various factors affecting false negatives.

Fetal cfDNA fraction is a key factor affecting the NIPT success rate. It is strongly influenced by gestational age at the initial blood sampling, maternal BMI, and maternal age (Kinnings et al., 2015; Qiao et al., 2019). For case 9, which was a twin pregnancy with advanced maternal age, the BMI and the fetal cfDNA fraction were 26.5 and 7.36%, respectively. Detection again failed at NIPT retesting with a fetal cfDNA fraction of 5.03%, but size-selection NIPT yielded a positive result. The twin pregnancy had a low fetal cfDNA fraction (<8%) (Suzumori et al., 2019) due to high BMI and advanced maternal age (Qiao et al., 2019). In addition, only one fetus had T18, so there was a much lower abnormal fetal cfDNA fraction in maternal total cfDNA, which lead to a false negative result with normal NIPT. Lo et al. (2010) reported that the main size distribution of maternal cfDNA was ∼20 bp longer than fetal cfDNA, suggesting that selective size selection of shorter cfDNA from maternal plasma could be an effective strategy to enrich fetal cfDNA (Fan et al., 2010). As shown in Figure 2, the cfDNA size peak distribution after E-gel-based size selection for case 9 was significantly shorter than that without size selection, and the fetal cfDNA fraction also significantly improved. This suggests that size-selection NIPT could increase the success rate of NIPT for women with twin pregnancies.

Fetal cfDNA in maternal peripheral blood originates from the trophoblast and mainly consists of placental DNA (Bianchi, 2004; Alberry et al., 2007). However, cases 7 and 8 were confirmed as CPM by placental tissue analysis; genetic discordance between placental and fetal tissues affects NIPT results and leads to false negatives (Pan et al., 2014). In further analysis, cases 7 and 8 showed negative results in retesting but had positive results with size-selection NIPT. Fetal DNA concentration analysis indicated that both cases had fetal cfDNA fractions >4% (Figure 2), but the fraction of abnormal cfDNA contained in maternal peripheral blood with CPM is significantly less than that for trisomies without CPM, which might explain the false negative results despite sufficient fetal cfDNA fraction. Size-selection NIPT could yield positive results for those CPM cases missed by normal NIPT by enriching abnormal fetal cfDNA concentrations. Based on the same theory, size-selection NIPT might be expanded to detect fetal copy number variants.

The initial NIPT for case 2 yielded a borderline unique reads number (<2 M), but positive results were achieved with retesting and size-selection NIPT. Placental tissues could not be collected for cases 3, 4, 5, and 6, so we were unable to confirm if those missed cases were due to CPM or other reasons. However, during prenatal screening, eight of the nine false negative cases showed multiple malformations on ultrasound, and those cases verified by invasive testing did have trisomies. A previous study reported that the nasal bone was hypoplastic/absent in 63.2% fetuses with chromosomal abnormalities (Viora et al., 2003). Therefore, cases with negative NIPT results but multiple malformation on ultrasound, especially absence of the nasal bone, should be given special attention and undergo invasive testing to avoid false negative results.

**FIGURE 3:**
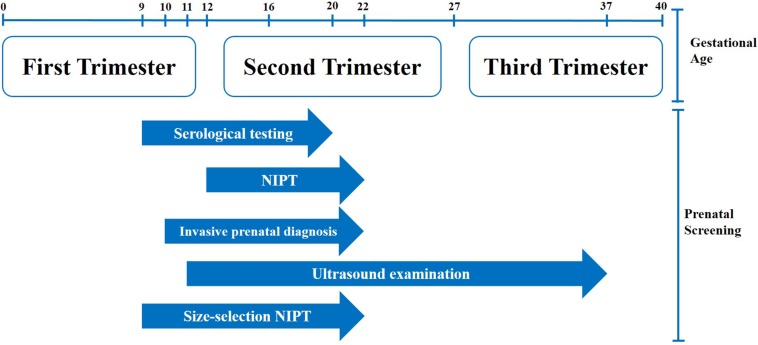
Prenatal diagnosis flowchart.

Serological testing is the primary prenatal screening method in China, while NIPT is mainly used to verify borderline positive cases after serological testing or those cases that cannot be screened by serological testing or invasive prenatal diagnosis. Fetal cfDNA fraction is lower in first trimester compared to the second trimester, and a low fetal cfDNA fraction will increase the NIPT false negative rate. The applicable gestational age for NIPT is from 12 to 22 weeks (Figure 3), and the subjects who underwent NIPT in our center had a median gestational age of 17 to 18 weeks. This meant that there was limited time to verify positive NIPT results in pregnant women with invasive prenatal diagnosis. However, size-selection NIPT could advancethe starting time point to be consistent with serological testing. Enriching the fetal cfDNA fraction and improving the NIPT success rate in first trimester would leave more time for invasive prenatal diagnosis for pregnant women with positive NIPT results.

He et al. (2018) reported 9 out of 11 false positive cases were categorized correctly as negative after size-selection NIPT. In Hu et al. (2019) also analyzed 1404 cases by magnetic bead-based size-selection NIPT, they found fetal cfDNA fraction increased from 11.3 ± 4.2 to 22.6 ± 6.6%, and 45.5% (6/11) false negative cases were detected after fetal cfDNA enrichment. In this study, we used E-Gel based size-selection NIPT, showed a 62.5% (5/8) success detection rate of false negative cases, which consist with the results of previous study (Hu et al., 2019), thus size-selection NIPT showed both higher sensitivity and specificity compared with traditional NIPT. However, fetal cfDNA fractions in the present study were selected with E-Gel EX 2% Gels after DNA library construction (He et al., 2018), which is a widely used *in vitro* size selection method (Mouliere et al., 2018). Compared with the E-Gel-based approach, a magnetic bead-based method could also be used for cfDNA size selection during DNA extraction (Minarik et al., 2015). Although this is more convenient, the range of cfDNA fragments obtained by magnetic beads may not be more accurate than the E-Gel-based method, thus resulted in a lower success detection rate compared with E-Gel-based method (45.5% vs. 62.5%) (Hu et al., 2019). Future work could compare the performance of both methods to choose the most appropriate one. In addition to physical size selection, virtual enrichment of the fetal cfDNA fraction in genomic data gained from the analysis of total cfDNA *in silico* size selection can also significantly improve NIPT sensitivity and specificity (Pan et al., 2014; Liu et al., 2019).

However, there are some limitations in this study. For example, only nine false negative cases were enrolled in this study, and the reason of false negative for some cases were not clear. Therefore, in further study, we will track more false negative cases to enhance our conclusion, and analyzed the samples by using more strategies.

## Conclusion

In conclusion, twin pregnancy and CPM result in lower abnormal fetal cfDNA fractions in maternal peripheral blood, while E-Gel-based cfDNA fragments size-selection NIPT could enrich the abnormal fetal cfDNA fraction and decrease the false negative rate of NIPT, even could bring forward of the start time of NIPT, and made it to be a primary method for prenatal screening.

## Data Availability Statement

The datasets for this article are not publicly available to assure patient privacy. Requests to access the datasets should be directed to TW, biowt@njmu.edu.cn.

## Ethics Statement

This study was approved by the Institutional Review Board of the Affiliated Suzhou Hospital of Nanjing Medical University. All subjects provided written informed consent prior to participation.

## Author Contributions

YX, GZ, BY, and TW: conception, design, collection, and assembly of data. JL: administrative support. YX, LQ, and JL: provision of study materials or patients. YX, GZ, LQ, BY, and TW: data analysis and interpretation. All authors: manuscript writing and final approval of manuscript.

## Conflict of Interest

The authors declare that the research was conducted in the absence of any commercial or financial relationships that could be construed as a potential conflict of interest.
